# Mainstreaming Gender-Responsive One Health: Now Is the Time

**DOI:** 10.3389/fpubh.2022.845866

**Published:** 2022-07-12

**Authors:** Julie Garnier, Sara Savić, Natalia Cediel, Paola Barato, Elena Boriani, Brigitte Bagnol, Richard Anthony Kock

**Affiliations:** ^1^Odyssey Conservation Trust, Bakewell, United Kingdom; ^2^Scientific Veterinary Institute “Novi Sad”, Novi Sad, Serbia; ^3^Observatorio Colombiano de Salud y Bienestar Animal, Facultad de Ciencias Agropecuarias, Universidad de La Salle, Bogotá, Colombia; ^4^CORPAVET and MolecularVet SAS, Bogotá, Colombia; ^5^EB Consult, Copenhagen, Denmark; ^6^Department of Anthropology, University of Witwatersrand, Johannesburg, South Africa; ^7^Department of Pathobiology and Population Sciences, Royal Veterinary College, London, United Kingdom

**Keywords:** gender, One Health, diseases, research, NEOH, policy, equity

## Introduction

The COVID-19 pandemic has unveiled the complex interconnectedness of human, animal, and environmental health and has epitomized the vital need to implement a One Health approach in order to address the multiple and inter-connected challenges faced by humanity today: biodiversity loss, climate change, and a health crisis signaling a new pandemic era. With an increasing awareness of the risk of abundant human and domestic animal populations providing opportunity for pathogens to emerge or re-emerge such as viruses (1, 2), a climate warming at unprecedented rates with extreme weather events breaking new records (3) and some ecosystems showing signs of “decompensation” as they can no longer ensure some of their vital functions (4), our future lies in rebuilding resilience through a One Health wisdom. One Health has been defined as “an integrated, unifying approach that aims to sustainably balance and optimize the health of people, animals and ecosystems” by the One Health High Level Expert Panel (OHHLEP) (5).

One Health has gained such momentum that an increasing number of academic and international institutions are integrating this concept in their strategic framework and curricula, as shown by the growing number of publications on this topic (6). One Health is also becoming a recurrent theme on the political agenda with the recent call by the G20 Health Ministers to “develop a joint WHO, OIE, FAO and UNEP strategy on One Health” (7). The recent creation of a One Health High Level Expert Panel to provide guidance on One Health-related matters that support cooperation among governments and to provide evidence-based scientific and policy advice to address the challenges raised by One Health, provides further political visibility to One Health (5).

But beyond adopting and implementing a One Health approach, policy-makers and stakeholders need to acknowledge that One Health will only be able to fulfill its vital purpose if some of the deep drivers of the inter-connected crises are addressed (8). A particularly neglected aspect in the One Health agenda is gender inequities, and there is an urgent need to unlock the full potential of women as agents of transformative change.

## Building Resilience Through Gender-Responsive One Health

Network for Ecohealth and One Health (NEOH) is the European chapter of Ecohealth International in which our Gender Working Group focuses on mainstreaming gender in One Health. Our group is mainly composed of women scientists with a strong experience in One Health who are actively involved in raising awareness on gender equity in One Health at all levels of society, in training stakeholders in integrating gender in their projects, in developing tools for mainstreaming gender in One Health research, training and in policy-making. But our lens goes beyond infectious diseases as we are looking at the interconnected deep drivers such as biodiversity loss, climate change, food insecurity, non-communicable diseases and other health challenges where gender inequities are prevalent. Recently, we developed a framework for mainstreaming a gender responsive and human-rights based One Health approach at all levels of society in order to protect nature, improve health and wellbeing and to prevent threats emerging at the Human/Animal/Environment interface. We reviewed gender roles, responsibilities and risks at the interface as well as gender and social inequities in the health, environment and climate spheres (9). By analyzing gender inequalities, roles, norms and power relations, we promote a “transformative” approach. We also reinforce norms that support gender equality and help create an enabling environment to improve the position of women, girls and marginalized groups, and to transform the underlying social structures, policies and norms that perpetuate gender inequalities.

The COVID-19 pandemic has deepened gender inequities by impacting women disproportionately, through an increased burden of unpaid care work, losses of jobs and livelihoods, a reduced access to sexual and reproductive health care services, an escalation in gender-based violence and an increasing risk of child marriage (10, 11). It has been estimated that the COVID-19 pandemic has set back gender equity by 36 years and that it will now take an average of 135.6 years for women and men to reach parity on a range of factors worldwide (12).

It is our opinion that we could build resilience better if a gender-responsive One Health is mainstreamed urgently.

## Individual, Household, and Community Level

Biologically, women are considered to be less at risk to develop most infectious diseases than men due to sex-based impact of hormonal and chromosomal control of immunity although pregnancy is a period of increased susceptibility to infections like brucellosis, listeriosis, toxoplasmosis and HIV infections (13–15) and to more severe illness with COVID-19 (16). But gender is a social determinant of health as gender norms, roles and relations influence people's susceptibility to health conditions and their access to health services for the prevention and control of infectious diseases. As a result, women are at higher risk and experience a more severe course of illness than men for many infectious diseases (17). Empowering women at the household and community level to participate in decision-making is therefore crucial to improve health and quality of life for women and their families, to reduce the burden of infectious diseases and to contribute to poverty reduction (18). Women with greater agency are more likely to access health services, including reproductive and maternal health, to have fewer children and their children are more likely to survive, receive better childcare at home and receive health care when they need it (19). Healthy women and girls can in turn more actively participate in society and take action to advance their own interests.

The recognition that gender needs to be integrated in infectious diseases management is not new. In 2011 WHO developed a gender framework for Emerging Infectious Diseases (20) but 10 years later it needs to be updated and to consider the full spectrum of sex and gender factors that influence disease vulnerability (21). In 2019 a Lancet Series on gender equality, norms and health highlighted all the relations between gender and health and the role of norms to improve health and equity (22). The gendered pathways of some infectious diseases such as Ebola are now well-established (23, 24) but mainstreaming gender-responsive analysis, surveillance, prevention and control plans are still desperately lacking for most infectious diseases. The deepening of gender inequities during the COVID-19 pandemic are a reflection of this great gap.

In a more integrated perspective, the importance of gender has long been acknowledged with water, sanitation, and hygiene (WASH) programmes in order to reduce the impact of diarrheal diseases and improve nutritional status, health and wellbeing, since in many resource poor settings women and girls are disproportionately responsible for water collection, cooking, cleaning and childcare (25). These integrated programmes also provide opportunities to move from “participation” to “power” in exploration of gender equality and to bridge practical gender needs (e.g., access to water) with gender interests (e.g., changes in power and roles) to achieve transformational changes in gender equality (26). The importance of women's contribution in protecting public health in Low-and Middle-Income Countries (27) and in bringing change for reaching the Sustainable Development Goals has been established (28). In Columbia, mothers' education beyond high school level was associated with higher immunization of children (29).

In the One Health/Ecohealth domain, gender issues have been described (30) and some networks have integrated gender modules in their training courses on infectious diseases, surveillance, response and control (31) but the operationalization of gender in One Health is still lagging behind, together with the integration of scientific and local knowledge.

## Academia and Research

Gender equality and mainstreaming in research have been established as priorities for the European Research Area since 2012 as an inclusive and diverse leadership helps bring forward new ideas and innovative approaches that better serve society (32), in addition to being a human rights issue. A lack of gender equality in leadership positions in research implies a considerable loss and waste of talent that detrimentally affects institutional decision-making by removing opportunities for women to shape and influence the research agenda (33). Some institutional changes have been encouraged with new comprehensive tools such as the GEAR (Gender Equality in Academia and Research) plan to implement gender equality plans in universities and research organizations (34) and innovative ways to enhance women participation in research fellowships (35). As a result, some positive trends were observed with almost gender parity at PhD graduate level in 2021 and a slight increase in the proportion of women holding the highest academic positions compared to previous years (32). But in reality, under-representation of women in senior academic and decision-making positions in the EU continues to be a significant issue. In 2018 women represented 40% of academic staff but only around a quarter (26.2%) occupied senior positions equivalent to full professorship, and the proportion of women as heads of institutions in the higher education sector stood at only 23.6% (32).

Strengthening gender equity in health systems actually requires the identification of the deep drivers of women under-representation using an intersectional approach, as well as involving experts in organizational psychology (organizational psychologists) in studying organizational drivers of under-representation (36–38) or gender analysis (9).

The importance of women's networks in promoting gender equity is highlighted by the Colombia Network of Scientific Women which helped to improve women's participation in science in this country, where women now represent 37.4 and 32.1% of scientists and research groups' leaders, respectively (39).

## Policy and Decision-Making Level

Gender relations of power constitute the root causes of gender inequality and are amongst the most influential of the social determinants of health. As a power relation, gender influences the nature of the health labor force, the implications of health financing, data collection and management, and how health policies and programmes are developed and implemented (19). Despite increasing evidence of the relationship between gender and health, inequities persist for example the persistent imbalance between the 70% of health workers who are female and the 70% of healthcare leaders who are male (22). Indirect impacts of such inequities can be dramatically violent. Recently health workers were involved in sexual abuse of vulnerable and under-aged girls during the course of interventions to managing the latest Ebola outbreaks in the DRC, with some cases of abuse being organized by networks of staff (33). Nearly $\frac{3}{4}$ of the workforce was occupied by males with a similar representation in leadership, highlighting the crucial need to offer equal space for women leadership in health and infectious disease management. Ensuring equal representation of women actually requires to develop strategies for negotiating difficult organizational cultures and political environments (40).

Women can also be powerful agents of change when in leadership positions in the climate and environmental domains, where positive outcomes will contribute to build resilience. Studies in India have found that women politicians were more likely to invest in public health infrastructure, and higher shares of women in parliament were associated with increased child immunizations and increased use of antenatal care (41). Correlations were found between women in positions of political authority and lower carbon footprints, between parliaments with greater proportions of female members and higher ratification of environmental treaties (42).

The equal representation of women at decision-making and policy-making level is therefore a fundamental aspect to build resilience and therefore be better prepared for future health crises including pandemics. An important step toward this goal has been made with the One Health High Level Expert Panel which shows gender parity among its 26 experts and which has adopted sociopolitical and multicultural parity as well as inclusion and engagement of communities and marginalized voices as one of its five underlying principles (5).

As One Health is now beginning to be taken into consideration in order to address the interconnected crises of pandemics, climate change and biodiversity loss, our opinion is that it will only be able to help shift the world onto a more resilient path if the deep drivers of gender inequities are addressed at all levels of society. Mainstreaming gender responsive and rights-based One Health will advance gender equality but gender equity will also enable to unlock the full potential of One Health.

## Author Contributions

JG and SS had the first idea of the topic and started the process. NC, PB, and EB contributed within their domain of research and RK and BB gave the final revision and addition to the text. All authors have contributed in writing the manuscript, we are all collaborating on the topic of Gender and One Health for some time.

## Conflict of Interest

EB was employed by EB Consult. The remaining authors declare that the research was conducted in the absence of any commercial or financial relationships that could be construed as a potential conflict of interest.

## Publisher's Note

All claims expressed in this article are solely those of the authors and do not necessarily represent those of their affiliated organizations, or those of the publisher, the editors and the reviewers. Any product that may be evaluated in this article, or claim that may be made by its manufacturer, is not guaranteed or endorsed by the publisher.
